# Supplementary material for: Differences in the dynamics of the tandem‐SH2 modules of the Syk and ZAP‐70 tyrosine kinases
Source: Protein Sci. 2021 Oct 23;30(12):2373–84. doi: 10.1002/pro.4199 (PMC8605373; doi:10.1002/pro.4199)
Supplement: Supplementary file 1 — Supplemental Table 1 Data Collection and Refinement Statistics [file PRO-30-2373-s003.docx]

Supplementary Material for:

**Differences in the dynamics of the tandem-SH2 modules of the Syk and ZAP-70 tyrosine kinases**

Helen T Hobbs,^1,2^ Neel H Shah,^3,4,6,7^ Jean M Badroos,^5^ Christine L Gee,^3,4^

Susan Marqusee,^1,3,5,6*^ and John Kuriyan^1,3,4,5,6*^

^1^ Department of Chemistry, University of California, Berkeley, United States.

^2^ Current address: Department of Biomedical Engineering, University of California, Irvine, United States

^3^ California Institute for Quantitative Biosciences, University of California, Berkeley, United States.

^4^ Howard Hughes Medical Institute, University of California, Berkeley, United States.

^5^ Department of Molecular and Cell Biology, University of California, Berkeley, United States.

^6^ Molecular Biophysics and Integrated Bioimaging Division, Lawrence Berkeley National Laboratory, Berkeley, United States.

^7^ Current address: Department of Chemistry, Columbia University, 3000 Broadway, New York, NY 10027

^*^Correspondence to John Kuriyan: 176 Stanley Hall, MC 3220, University of California, Berkeley, CA, 94720, USA. Phone: (510) 643-1710. Fax: (510) 643-2352. Email: [kuriyan@berkeley.edu](mailto:kuriyan@berkeley.edu)

^*^Correspondence to Susan Marqusee: 576 Stanley Hall, University of California, Berkeley, CA, 94720, USA. Phone: (510) 642-7678. Email: [marqusee@berkeley.edu](mailto:marqusee@berkeley.edu)

**SUPPLEMENTARY MATERIALS AND METHODS**

*Structure determination of the isolated ITAM-free Syk tSH2 module*

Data was collected at the Advanced Light Source at the Lawrence Berkeley National Laboratory at λ= 0.999816 Å over 402°, with Δφ=1° frames and an exposure time of 1 s per frame. The proper range for data collection was determined using iMOSFLM.^1^ Data were processed using with XDS and then scaled and merged with Aimless in the CCP4 suite.^2–4^ Molecular replacement was performed using Phenix Phaser using Chain F from the ITAM-bound structure as a search model (PDB: 1A18).^5,6^ Refinement was performed using Phenix, manual refinement was done using Coot, and model geometry was assessed using Molprobity.^5–11^ Coordinates and structure factors were deposited in the Protein Data Bank, with accession code 7SA7.

*HDX-MS methods*

For both the ITAM bound and ITAM-free states, a tandem mass spec experiment was performed on undeuterated samples in order to sequence and identify peptides. Byonic (Protein Metrics) was used to generate peptide lists based on the tandem MS experiment. All samples were thawed immediately before injection into an LC system (Trajan and Thermo). The temperature of the LC columns was maintained at 4 ^o^C in a cooled chamber in order to reduce back exchange. The quenched sample was subjected to an in-line digestion by two acid proteases, pepsin and fungal protease (Sigma). Following digestion, peptides were de-salted on a C4 trap column before analytical separation and elution with a 10-50% and then short 100% wash with 90% acetonitrile on a C8 analytical column. Peptides were eluted directly into a Q-Exactive Orbitrap mass spectrometer for analysis. Peptide deuteration states were determined by HD Examiner 3 (Sierra Analytics) by fits of the isotopic distribution to those predicted from the tandem mass spec experiment. Deuteration of peptides here is reported as #D incorporated, as calculated by HD Examiner 3. The full HDX data set for Syk and ZAP-70 can be found in the source materials.

*MD simulations-calculation of RMSF*

The AmberTools CPPPTRAJ package was used to analyze the simulations.^12^ The distance between the SH2 domains was calculated as the distance between the center of mass of the N-terminal SH2 domain (residues 8-110) and the center of mass of the C-terminal SH2 domain (residues 161-252).

We computed the root-mean-square fluctuation (RMSF) for each domain simulation by first determining its average structure across the simulation, after removing the first 30 nsec to ensure the simulation had sufficient time to equilibrate, and then calculating the RMSF from this average structure. The following residue numbers were used to align the Cα atoms of each domain in Syk: N-SH2 (9-110), C-SH2 (162-253), and inter-SH2 linker (111-161); and in ZAP-70: N-SH2 (9-110), C-SH2 (162-253), and inter-SH2 linker (111-161). The error bars depicted on the graphs of the RMSF refer to the standard deviation of RMSF values for each simulation, divided by the square root of the number of simulations per condition, or the standard error. Finally for the distance between the beta strands was calculated as the difference between the carbonyl oxygen of residue 212 and the backbone nitrogen of residue 197 for ZAP-70 and the carbonyl oxygen of residue 211 and the backbone nitrogen of residue 196 for Syk.

**Supplemental Table 1.** Data Collection and Refinement Statistics

|  | **ITAM-free Syk tSH2 module** |
| --- | --- |
| **Wavelength** |  |
| **Resolution range** | 45.78 - 3.2 (3.314 - 3.2) |
| **Space group** | C 1 2 1 |
| **Unit cell** | 143.882 153.881 85.472 90 91.049 90 |
| **Total reflections** | 61287 (6053) |
| **Unique reflections** | 30660 (3029) |
| **Multiplicity** | 2.0 (2.0) |
| **Completeness (%)** | 99.67 (99.11) |
| **Mean I/sigma(I)** | 7.02 (0.98) |
| **Wilson B-factor** | 73.77 |
| **R-merge** | 0.1197 (0.8567) |
| **R-meas** | 0.1693 (1.212) |
| **R-pim** | 0.1197 (0.8567) |
| **CC1/2** | 0.985 (0.367) |
| **CC*** | 0.996 (0.733) |
| **Reflections used in refinement** | 30597 (3018) |
| **Reflections used for R-free** | 1588 (167) |
| **R-work** | 0.2697 (0.3392) |
| **R-free** | 0.3039 (0.3440) |
| **CC(work)** | 0.916 (0.607) |
| **CC(free)** | 0.872 (0.682) |
| **Number of non-hydrogen atoms** | 11300 |
| **macromolecules** | 11296 |
| **solvent** | 4 |
| **Protein residues** | 1427 |
| **RMS(bonds)** | 0.002 |
| **RMS(angles)** | 0.67 |
| **Ramachandran favored (%)** | 95.66 |
| **Ramachandran allowed (%)** | 3.84 |
| **Ramachandran outliers (%)** | 0.50 |
| **Rotamer outliers (%)** | 9.04 |
| **Clashscore** | 9.07 |
| **Average B-factor** | 77.49 |
| **macromolecules** | 77.51 |
| **solvent** | 42.73 |

Statistics for the highest-resolution shell are shown in parentheses.

**REFERENCES**

1. Battye TGG, Kontogiannis L, Johnson O, Powell HR, Leslie AGW (2011) iMOSFLM: a new graphical interface for diffraction-image processing with MOSFLM. Acta Crystallogr D Biol Crystallogr 67:271–281.

2. Kabsch W (2010) XDS. Acta Crystallogr D Biol Crystallogr 66:125–132.

3. Evans PR, Murshudov GN (2013) How good are my data and what is the resolution? Acta Crystallogr D Biol Crystallogr D69:1204–1214.

4. Winn MD, Ballard CC, Cowtan KD, Dodson EJ, Emsley P, Evans PR, Keegan RM, Krissinel EB, Leslie AGW, McCoy A, McNicholas SJ, Murshudov GN, Pannu NS, Potterton EA, Powell HR, Read RJ, Vagin A, Wilson KS (2011) Overview of the CCP4 suite and current developments. Acta Cryst D67:235–242.

5. McCoy AJ (2007) Solving structures of protein complexes by molecular replacement with Phaser. Acta Crystallogr D Biol Crystallogr 63:32–41.

6. Adams PD, Afonine PV, Bunkóczi G, Chen VB, Davis IW, Echols N, Headd JJ, Hung L-W, Kapral GJ, Grosse-Kunstleve RW, et al. (2010) PHENIX: a comprehensive Python-based system for macromolecular structure solution. Acta Crystallogr D Biol Crystallogr 66:213–221.

7. Emsley P, Lohkamp B, Scott WG, Cowtan K (2010) Features and development of Coot. Acta Crystallogr D Biol Crystallogr 66:486–501.

8. Chen VB, Arendall WB, Headd JJ, Keedy DA, Immormino RM, Kapral GJ, Murray LW, Richardson JS, Richardson DC (2010) MolProbity: all-atom structure validation for macromolecular crystallography. Acta Crystallogr D Biol Crystallogr 66:12–21.

9. Laskowski RA, Moss DS, Thornton JM (1993) Main-chain bond lengths and bond angles in protein structures. J Mol Biol 231:1049–1067.

10. Vaguine AA, Richelle J, Wodak SJ (1999) SFCHECK: a unified set of procedures for evaluating the quality of macromolecular structure-factor data and their agreement with the atomic model. Acta Crystallogr D Biol Crystallogr 55:191–205.

11. Berman H, Henrick K, Nakamura H (2003) Announcing the worldwide Protein Data Bank. Nat Struct Biol 10:980.

12. Roe DR, Cheatham TE (2013) PTRAJ and CPPTRAJ: Software for Processing and Analysis of Molecular Dynamics Trajectory Data. J. Chem. Theory Comput. 9:3084–3095.

**SUPPLEMENTARY MATERIALS FIGURE LEGENDS**

***Figure S1.***  *The ITAM-free tSH2 module of ZAP-70 is more dynamic than that of Syk*

The C- SH2 domain of ZAP-70 and a section of the inter-SH2 linker are more dynamic than the corresponding regions in Syk as measured by the root-mean-square-fluctuation (Å) of each Cα atom in the individual domain about its average position in that domain across five independent simulations of each of the ITAM-free tSH2 modules. Error bars represent the SEM (n=5).

***Figure S2.*** *Central β-strands* *in the C-SH2 of ZAP-70 fluctuate throughout the simulations.* The distance (Syk in red, ZAP-70 in blue) between two of the β-strand residues each pair of simulations. These β-strands in ZAP-70 move apart to a greater extent and this separation is sustained for longer than in Syk in most of the simulations.
