# Supplementary figures and images for: Differences in the dynamics of the tandem‐SH2 modules of the Syk and ZAP‐70 tyrosine kinases
Source: Protein Sci. 2021 Oct 23;30(12):2373–84. doi: 10.1002/pro.4199 (PMC8605373; doi:10.1002/pro.4199)

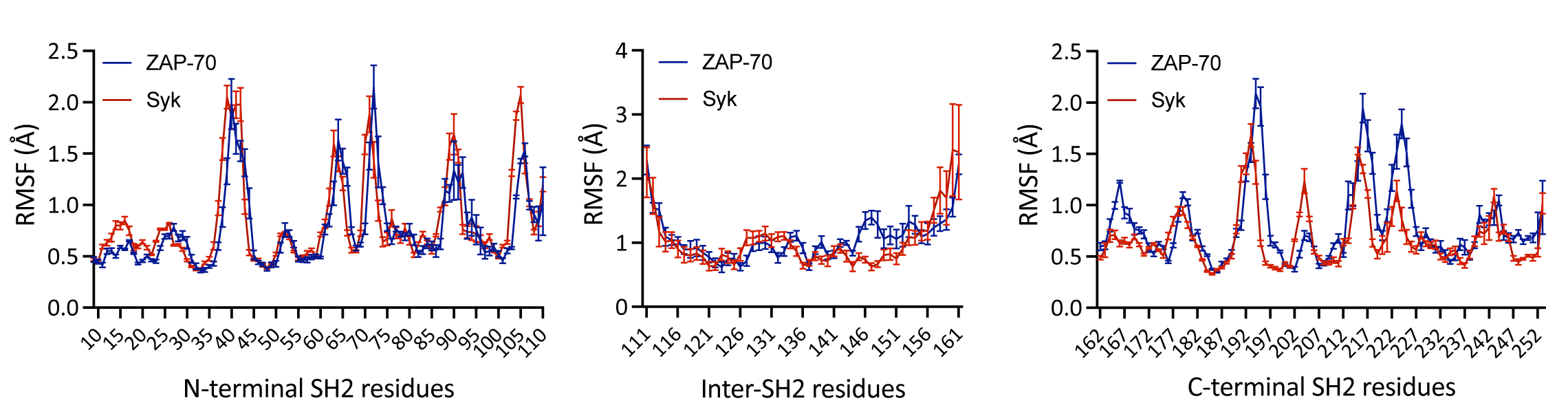

Supplement: Supplementary file 2 — Figure S1 The ITAM‐free tSH2 module of ZAP‐70 is more dynamic than that of Syk. The C‐ SH2 domain of ZAP‐70 and a section of the inter‐SH2 linker are more dynamic than the corresponding regions in Syk as measured by the root‐mean‐square‐fluctuation (Å) of each Cα atom in the individual domain about its average position in that domain across five independent simulations of each of the ITAM‐free tSH2 modules. Error bars represent the SEM (n = 5). [file PRO-30-2373-s002.tif]

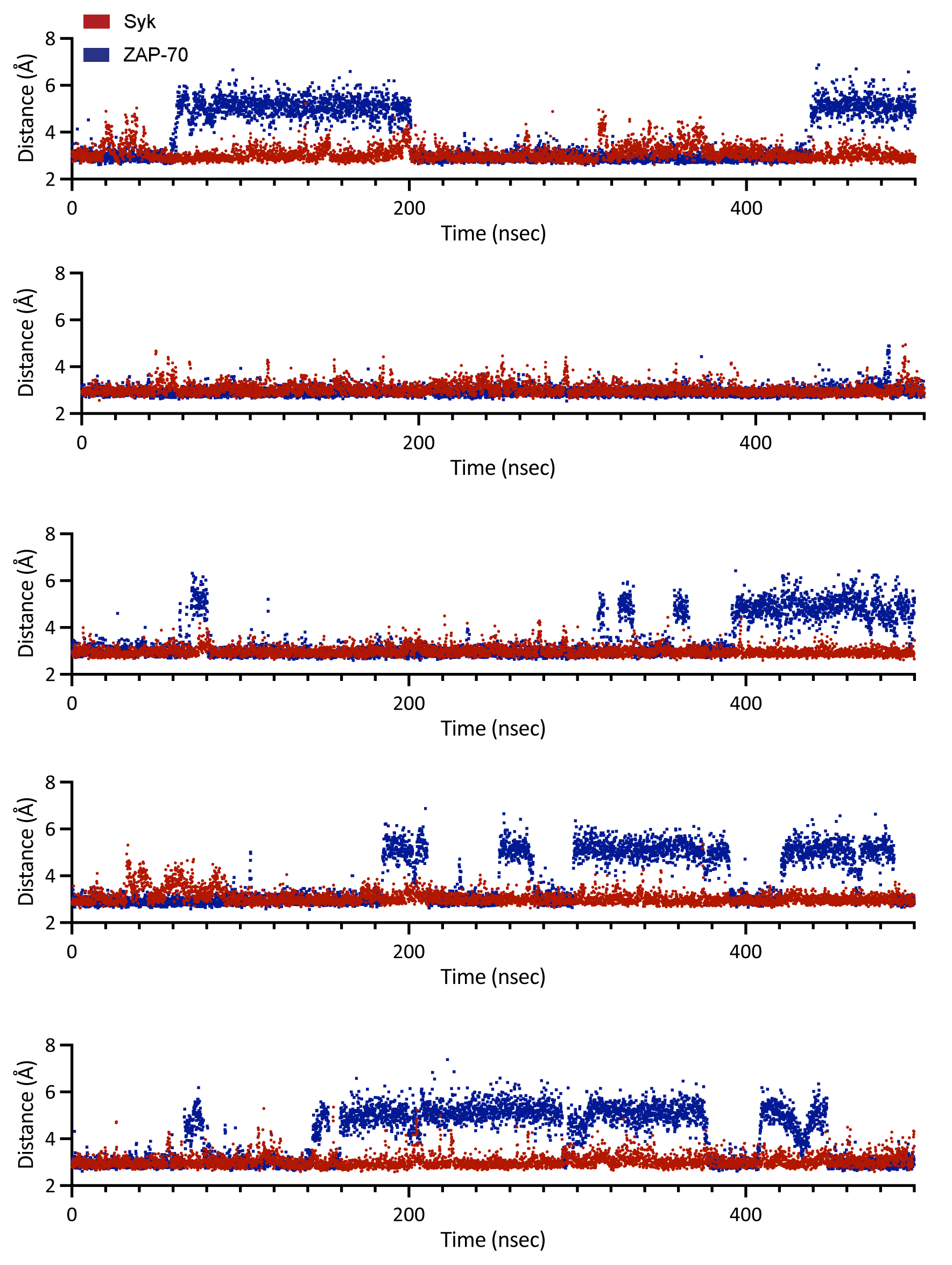

Supplement: Supplementary file 3 — Figure S2 Central β‐strands in the C‐SH2 of ZAP‐70 fluctuate throughout the simulations. The distance (Syk in red, ZAP‐70 in blue) between two of the β‐strand residues each pair of simulations. These β‐strands in ZAP‐70 move apart to a greater extent and this separation is sustained for longer than in Syk in most of the simulations. [file PRO-30-2373-s001.tif]
